# Editorial: Novel diagnostic and therapeutic strategies in the management of cerebral gliomas

**DOI:** 10.3389/fonc.2022.1115410

**Published:** 2023-01-12

**Authors:** Maria Caffo, Letteria Minutoli, Emeline Tabouret, Valeria Barresi

**Affiliations:** ^1^ Department of Biomorphology and Dental Sciences and Morphofunctional Imaging, Unit of Neurosurgery, University of Messina, Messina, Italy; ^2^ Department of Biomorphology and Dental Sciences and Morphofunctional Imaging, University of Messina, Messina, Italy; ^3^ Neuro-Oncology, Assistance publique Hôpitaux de Marseille (APHM), Timone Hospital, Aix-Marseille Université, Marseille, France; ^4^ Department of Diagnostics and Public Health, Section of Anatomic Pathology, University of Verona, Verona, Italy

**Keywords:** Glioma, Target-therapy, EGFR receptor, awake surgery, tumor progression monitoring

Gliomas are the most common tumors in the central nervous system (CNS). Among these, glioblastoma (GBM) IDH-wildtype is the most frequent, accounting for about 14% of all primary neoplasia involving the CNS (1). The current gold standard treatment for adult patients with high-grade gliomas, including GBM IDH-wildtype, is surgery followed by concurrent radiotherapy and temozolomide chemotherapy (2). However, this treatment has limited efficacy and patients inevitably develop disease recurrence and poor outcome. Over the last decade, there has been increasing knowledge of the molecular alterations of gliomas, clarifying that the prognosis of these tumors largely depends on their epigenetic and genetic profile, rather than on their histopathological features. Therefore, tumors with overlapping morphology may have different clinical outcomes because they are genetically different. The increasing knowledge of the molecular landscape of high-grade gliomas has strongly contributed to the development of therapies alternative or complementary to temozolomide for treatment of these neoplasia. Approximately 50% of IDH-wildtype GBMs harbor epidermal growth factor receptor (EGFR) amplification, and consequent EGFR overexpression, suggesting the potential efficacy of EGFR-inhibitors in this subgroup. However, ocular side effects were reported in patients with EGFR- amplified IDH-wildtype GBMs treated with the EGFR inhibitor depatuxizumab mafodotin (ABT-414) (Parrozzani et al.). These adverse effects are likely related to the role of EGFR and its ligand EGF in the maintenance of the normal thickness of corneal epithelium and in its post- wound healing. In spite of their reversibility, their prolonged persistence and influence of adverse effects on the patient’s quality of life should be considered when using this treatment. Approximately 0.56-1.69% of GBMs in the adult display neurotrophic tyrosine receptor kinase (NTRK) fusions as the driver genetic alteration and could be treated using NTRK inhibitors (Wang et al.). However, it should be mentioned that the efficacy of several chemotherapeutic agents, successfully used for many peripheral cancers, could be dramatically reduced in patients with gliomas because of poor penetration of the blood-brain-barrier (BBB). In this regards, several strategies have been explored to overcome the limitations of the BBB in the treatment of brain tumors, including the use of carrier molecules that are permeable to the barrier (Cooper et al.). Because of their high tumor specificity and biocompatibility, heptamethine cyanine dyes (HMCDs) have recently been proposed as a potential drug delivery system that can overcome the challenges surrounding BBB penetration.

A major issue in the follow-up of glioma patients during or after standard treatments is the distinction between tumor progression and pseudo-progression. This latter is a treatment effect that typically occurs 3-6 months after chemoradiotherapy and that can mimic tumor progression. Dosage of circulating tumor biomarkers, such as circulating tumor cells, tumor DNA or extracellular vesicles- derived biomarkers, may be potentially useful in distinguishing between these two conditions. However, according to a recent meta-analysis on this theme, no biomarkers are yet ready for clinical application as triage tests or as add-on tests to the current GBM monitoring paradigm and further assay refinement and evaluation in larger cohorts with diagnostic accuracy study designs are required. In patients treated with immunotherapy, inflammatory response may mimic tumor progression at imaging, because it produces a volumetric increase in the tumor contrast-enhanced areas. In these cases, surgical resection of the lesion followed by single-cell RNA sequencing study of the tumor microenvironment may be useful to assess tumor response to therapy and to assist with clinical decision-making (Raza et al.).

In this genomic era, several studies also demonstrated the still prominent role of surgery in determining the clinical outcome of gliomas. For instance, in a cohort of 326 patients with IDH- wildtype GBM, extent of resection of the contrast-enhanced tumor > 97% and of the non-enhanced tumors ≥ 30% was associated with a significant increase in the overall and progression-free survival (Incekara et al.). However, maximal tumor resection may be hampered by tumor size or location in anatomical sites difficult to be approached. For instance, giant insular gliomas are usually not amenable to complete surgical resection and are associated with significant post-operative morbidity (Rossi et al.). Nonetheless, the use of a transcortical approach with extensive brain mapping under awake anesthesia may reduce the post-operative morbidity and allow a higher extent of resection in the meantime, as shown in a retrospective analysis on 95 patients (Rossi et al.). In this setting, the tumor infiltration of deep perforating arteries represents the most relevant risk factor associated with long-term neurological sequelae after surgery (Rossi et al.).

Hyperthermia has recently emerged as a potential alternative therapeutic approach for cancer patients (3). This is based on the concept that high temperatures may lead to irreversible cell injury in target regions and that cancer tissues are more thermosensitive than normal tissues (3). The potential application of nanoparticle (NP)-mediated plasmonic photothermal therapy for GBM has been assessed *in vitro* and in preclinical studies *in vivo* (Bastiancich et al.). Major challenges in the clinical translation of this therapeutic strategy are that NPs should pass the BBB to reach the tumor site at therapeutic concentrations, that they should be selectively uptake in tumor cells with sparing of healthy cells, and that heating should be sufficient to induce cell injury while avoiding brain damage (Bastiancich et al.). The valuable manuscripts published in this Research Topic largely contributed to highlight recent diagnostic and therapeutic advances in gliomas. We hope that the presented issues will drive further experimental research leading to an improved clinical outcome in glioma patients in the future.

## Author contributions

MC and VB conceived and wrote the draft. LM and ET revised the manuscript in the light of bibliographic references. All authors contributed to the article and approved the submitted version.
